# Identifying Best Practices for WISEWOMAN Programs Using a Mixed-Methods Evaluation

**Published:** 2005-12-15

**Authors:** Melanie Besculides, Heather Zaveri, Rosanne Farris, Julie Will

**Affiliations:** Mathematica Policy Research, Inc; Mathematica Policy Research, Inc, Princeton, NJ; Centers for Disease Control and Prevention (CDC), Atlanta, Ga; CDC, Atlanta, Ga

## Abstract

**Introduction:**

Recommendations on best practices typically are drawn from unique settings; these practices are challenging to implement in programs already in operation. We describe an evaluation that identifies best practices in implementing lifestyle interventions in the Center for Disease Control and Prevention's WISEWOMAN program and discuss our lessons learned in using the approach.

**Methods:**

We used a mixed-methods evaluation that integrated quantitative and qualitative inquiry. Five state or tribal WISEWOMAN projects were included in the study. The projects were selected on the basis of availability of quantitative program performance data, which were used to identify two high-performing and one low-performing site within each project. We collected qualitative data through interviews, observation, and focus groups so we could understand the practices and strategies used to select and implement the interventions. Data were analyzed in a multistep process that included summarization, identification of themes and practices of interest, and application of an algorithm.

**Results:**

Pilot testing data collection methods allowed for critical revisions. Conducting preliminary interviews allowed for more in-depth interviews while on site. Observing the lifestyle intervention being administered was key to understanding the program. Conducting focus groups with participants helped to validate information from other sources and offered a more complete picture of the program.

**Conclusion:**

Using a mixed-methods evaluation minimized the weaknesses inherent in each method and improved the completeness and quality of data collected. A mixed-methods evaluation permits triangulation of data and is a promising strategy for identifying best practices.

## Introduction

There is no doubt that public health programs should follow best practices. In the programmatic setting, best practices are the processes that lead to the implementation of the most appropriate intervention for a given location and population (1). Identifying and applying best practices is complex — largely because recommendations on what works are based typically on experimental or other one-of-a-kind settings. As a result, the practices recommended are not likely to be relevant to most other settings. An alternative is to identify best practices by collecting data from existing programs and to use two or more complementary methods, or a mixed-methods approach, to data collection (2). A mixed-methods approach can be a combination of one or more qualitative methods or a mix of qualitative and quantitative methods.

A mixed-methods approach strengthens evaluation research, because no single method is without weakness or bias (3). Quantitative data, for example, may be objective, but they often lack the depth needed to elucidate how and why a program works. Qualitative data can enhance understanding of program implementation and operation, but are considered less objective. By combining the two, research can be both objective and rich.

There are several qualitative methods, each with strengths and weaknesses. For instance, although interviews with program staff can provide a detailed picture of program operations, they cannot objectively provide the range of participants' perspectives (T.S. Weisner, personal communication, September 2005). Focus group participants can provide information on program experiences and effects, but this information is not generalizable because focus group members typically do not represent all program participants.

This article describes a mixed-methods approach to identifying best practices in the Centers for Disease Control and Prevention's (CDC's) WISEWOMAN (Well Integrated Screening and Evaluation for Women Across the Nation) program.

In 1995, the CDC created the WISEWOMAN program, which was authorized by Congress in the Breast and Cervical Cancer Mortality Prevention Act of 1990 (Public Law 101-354). WISEWOMAN is designed to build upon the National Breast and Cervical Cancer Early Detection Program (NBCCEDP) by offering 1) screening for risk factors associated with cardiovascular disease and 2) lifestyle intervention services to women aged 40 to 64 years who participate in NBCCEDP. WISEWOMAN participants must be uninsured and ineligible for Medicaid.

Although the cardiovascular screening is undoubtedly important, the lifestyle intervention offered through WISEWOMAN is a key service intended to modify the behaviors associated with increased risk for cardiovascular and other chronic diseases. In fact, the intervention is predicated on the notion that obesity, poor diet, physical inactivity, and tobacco use can be modified to reduce high blood pressure and elevated serum cholesterol levels at relatively low cost and with minimal risk to participants. Theoretically, a reduction in risk factors leads to a decreased incidence of cardiovascular events such as myocardial infarction. The literature on lifestyle interventions suggests that a combination of diet and physical activity is most effective in reducing the risk factors for cardiovascular disease in women (4,5).

The CDC not only requires all WISEWOMAN programs to offer a lifestyle intervention but also encourages them to use the national guidelines for heart-healthy eating, physical activity, and tobacco cessation in developing their interventions (6). Beyond this, the CDC does not prescribe the lifestyle intervention, preferring instead to have projects develop or select a culturally appropriate intervention shown by scientific evidence to be effective either in lowering blood pressure or cholesterol levels or in improving diet and physical activity (6). Lifestyle interventions therefore vary among states and tribes. Although state or tribal programs often dictate which intervention should be used at their local sites, they sometimes allow flexibility in how sites implement the intervention. To distill a set of best practices from these highly variable interventions, Mathematica Policy Research, Inc (MPR) reviewed the literature on lifestyle interventions and collected qualitative data from sites through interviews, observation, and focus groups. The best practices identified will be disseminated to existing and new WISEWOMAN practitioners through a user-friendly toolkit.

## Methods

We conducted in-depth case studies of selected WISEWOMAN projects and of high-performing and low-performing local sites within each project. Case studies allowed us to explore how and why projects and local sites used certain practices, providing insight into the relationship between program implementation and program effectiveness (7). The practices of high-performing sites were compared with practices of low-performing sites to identify if and how they differed. To conduct this mixed-methods evaluation, we applied the following five steps: 1) site selection using quantitative program performance data, 2) development of a conceptual framework for guiding qualitative inquiry, 3) development and refinement of data collection instruments, 4) collection of qualitative data, and 5) analysis of qualitative data to identify best program practices.

We applied the RE-AIM (Reach, Effectiveness, Implementation, Adoption, and Maintenance) model, developed by Glasgow et al (8), as an organizing framework for our study. The purpose of the model is to facilitate evaluations of the translatability and overall public health impact of a health promotion intervention (8). The framework specifies dimensions at the individual and institutional levels. Dimensions are defined as the following: 1) the intervention's *reach* into the target population, 2) its *effectiveness* in modifying risk, 3) its *adoption* by target settings, 4) its consistent *implementation*, and 5) *maintenance* of its effects among participants and target settings (8). Including both the individual and institutional levels in the framework allows an investigation of whether an intervention has an impact on one level but not the other. Both are essential in determining which interventions will work in other settings and have a strong impact on overall public health.

### Step 1: site selection

The first step of this evaluation was to select state or tribal projects and identify the highest-performing and lowest-performing local sites within each project using the RE-AIM framework. Fifteen state or tribal WISEWOMAN projects are currently funded; each has been operating for a different length of time. Through a separate contract, the CDC worked with the Research Triangle Institute (RTI) to select five projects for the MPR study. The five projects selected had program data for at least 100 women per local site, which allowed the tracking of participants from the time of program enrollment through a rescreening 10 to 14 months later.

**Figure 1:**
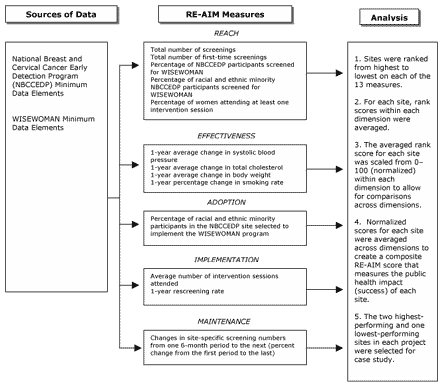
Site selection method for WISEWOMAN study on best practices using the RE-AIM framework. RE-AIM indicates Reach, Effectiveness, Adoption, Implementation, and Maintenance (8). [A full-page version of this figure is available in PDF format (166K)]

The CDC and RTI then developed a method for rating performance along RE-AIM dimensions for three local sites — the two highest-performing and one lowest-performing — from each of the five projects, providing a total of 15 sites for case studies (R.P. Farris, J. Will, O. Khavjou, E.A. Finkelstein, unpublished data, 2004). MPR and a WISEWOMAN consultant group provided input on site selection methods. Figure 1 identifies the data elements and measures that were used to calculate the RE-AIM scores for site selection. For example, *reach* is measured with 1) total number of screenings, 2) total number of women screened for the first time, 3) percentage of NBCCEDP participants screened for WISEWOMAN, 4) percentage of minority NBCCEDP participants screened for WISEWOMAN, and 5) percentage of women attending at least one intervention session. Effectiveness is measured with 1-year average changes in 1) systolic blood pressure, 2) total cholesterol, 3) body weight, and 4) percentage change in smoking rate.

**Figure 2:**
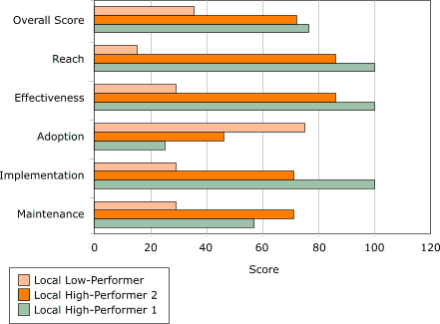
Overall RE-AIM score (an average of five RE-AIM scores) and individual RE-AIM scores for each of three local sites within one WISEWOMAN project. RE-AIM indicates Reach, Effectiveness, Adoption, Implementation and Maintenance (8)

Program data from each project were analyzed separately, and each site's performance was ranked as follows: first, sites were ranked from highest to lowest on each of five RE-AIM dimensions. For every site, the rank scores within each dimension were averaged. Then, within each dimension, the averaged rank score for each site was scaled from 0 to 100 (normalized) to allow for comparisons across dimensions. Normalized scores for each site were then averaged across dimensions to create a composite RE-AIM score that measures the overall public health impact (success) of each site (Figure 2). The figure also illustrates the resulting normalized rank score for each dimension in three local sites from one project. After the one low-performing and two high-performing sites were selected for a project, researchers were given the names of the sites but not their rank. This approach ensured that rank did not influence perception of local sites during qualitative data collection and initial data analysis.

**Table:** 

### Step 2: development of conceptual framework for qualitative inquiry

The second step was to develop a conceptual framework for guiding qualitative inquiry. The overarching research questions for qualitative data collection were:

- What are the most effective practices used in selected state or tribal WISEWOMAN projects and local sites to design and deliver lifestyle interventions?
- How have projects and local sites implemented these practices?
- What lessons can these practices offer to other projects and local sites?

**Figure 3:**
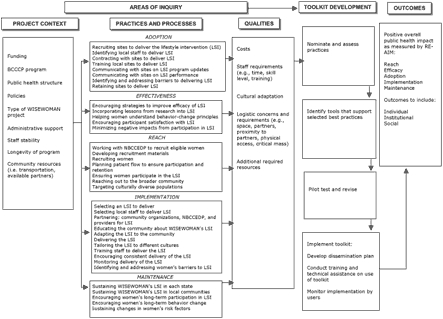
Framework for guiding qualitative inquiry for WISEWOMAN study on best practices. [A full-page version of this figure is available in PDF format (454K)]

Figure 3 depicts the framework that addresses these questions.

### Step 3: development and refinement of data collection instruments 

The next step was to develop a data collection plan and data collection instruments based on the framework for qualitative inquiry. The instruments were piloted in one project to assess how well they captured data. They were then revised to focus more on the lifestyle intervention rather than the broad program-implementation processes.

The three objectives of the qualitative data collection plan were as follows:

- To gather enough information about selected WISEWOMAN projects and local sites to understand the contexts in which programs operate, the design and implementation of different lifestyle interventions, and how each intervention fits into the overall approach to service delivery in each project or site
- To gather detailed qualitative information on practices related to the delivery of the lifestyle intervention and the five RE-AIM dimensions
- To systematically assess the most effective practices according to each RE-AIM dimension

To reach these objectives, we developed semistructured interview guides for each type of informant: federal project officers, state or tribal project directors and coordinators, and local coordinators, interventionists, and partners. We also developed a guide for focus groups, which were composed of program participants, and for observation of lifestyle intervention sessions, which also involved program participants. Each guide was organized by a RE-AIM dimension to ensure that data were collected systematically and consistently across projects and local sites. Although the interview guide provided questions, the order of the questions was not prescribed to researchers conducting the interviews, and the questions were not intended to be asked verbatim. Figure 4 shows the interview topics that were included in all guides. All methods involving program participants (i.e., focus groups and observations) were approved by the institutional review board (IRB) of Public/Private Ventures (Philadelphia, Pa).

**Figure 4:** Semistructured interview guide topics for WISEWOMAN study on best practices. Each of the five RE-AIM dimensions are identified in parentheses. RE-AIM indicates Reach, Effectiveness, Adoption, Implementation, and Maintenance (8). Not all interviews included all questions. Order of questions was not prescribed; order here reflects a logical interview flow.

1. Background Information on Interview Participant

2. Overview of the WISEWOMAN Projects

- Overall structure at state and local levels
- Chronology and development

3. Local Site Involvement *(Adoption)*

- Recruitment strategies and contracting mechanisms with local sites
- Common barriers to site participation and how they have been addressed

4. Participant Recruitment and Involvement *(Reach)*

- Process for recruiting participants into WISEWOMAN and the lifestyle intervention
- Initiatives for targeting specific groups of women for participation

5. Design and Delivery of the Lifestyle Intervention *(Implementation)*

- Overall design of the intervention
- Feasibility and consistency of intervention delivery
- Staffing issues, including commitment, availability, training, and others

6. WISEWOMAN'S Effect on Participants *(Effectiveness)*

- Perceived effectiveness of the lifestyle intervention
- Impact on participants' short-term and long-term quality of life

7. Program Sustainability *(Maintenance)*

- Challenges to ongoing service delivery
- Strategies for overcoming challenges both at state and local levels

8. Additional Critical Themes and Wrap-up

- Role of key community and other partners

### Step 4: qualitative data collection

Qualitative data collection consisted of preliminary data collection and site visits. The goal of preliminary data collection was to understand the context in which selected projects operated and learn how projects or sites deliver the lifestyle intervention to WISEWOMAN participants. Data were derived from a review of project and local site reports and from semistructured telephone interviews with CDC staff members who oversee the study projects, state or tribal project staff, and staff at each local site.

Site visits were conducted by two researchers at each state or tribal project and at the three selected local sites within each project. The purpose of the visits was to expand on what we learned from the preliminary data about how lifestyle interventions are developed and implemented. Toward this end, we conducted individual and small group interviews with staff and focus groups with current program participants; we also observed lifestyle intervention sessions.

#### Interviews

In-person interviews were conducted with staff members who play a role in developing or delivering the lifestyle intervention at the project and site levels. When possible, we also interviewed staff of local program partners, such as a community swimming pool that offers discounted passes to WISEWOMAN participants as a way to increase their physical activity. The goal of the interviews was to learn about the staff's perspectives on practices used to implement the lifestyle intervention. We also gathered data to *triangulate*, or to combine and compare responses from multiple informants and sources, to develop a consistent understanding of lifestyle intervention implementation.

#### Focus groups

We conducted a focus group with WISEWOMAN participants at each local site to better understand the intervention's reach, effectiveness, implementation, and ability to maintain behavioral change. When recruiting women for the focus group, we attempted to select those who had participated in the lifestyle intervention recently and could address why they chose to participate, as well as women who participated more than a year ago and could address maintenance of the intervention. Each woman was given a $25 incentive to participate in the focus group. We typically invited 10 to 12 women to attend each focus group, which resulted in six to nine participants.

#### Observations

We developed procedures for observing various forms of intervention delivery, including individual in-person, telephone, and group interventions. Before researchers were permitted to observe the sessions, the interventionist solicited the participants' consent — by obtaining either a signature (for in-person and group interventions) or verbal consent (for telephone interventions). For scheduling purposes, we restricted our observations to what was scheduled the day we visited the site, so we did not know in advance the type of intervention we would observe (i.e., an initial visit or a follow-up visit).

### Step 5: Qualitative data analysis to identify best program practices

This analysis consisted of four steps: writing site reports, developing theme tables, identifying practices of interest, and applying a best-practices algorithm.

#### Writing site reports

Both members of the site visit team worked together to write a report for each state or tribal project, for each local site within a project, and for each data source (e.g., interviews, focus groups). The rationale for joint report writing was to minimize the degree to which one researcher's impressions might influence the findings. In addition, to ensure that the reports were accurate, they were reviewed by interviewees at the state or tribal projects and local sites. To protect confidentiality, focus group data were not shared with program staff.

#### Developing theme tables

Theme tables, developed from the reports, were intended to present data in a simplified manner grouped by theme. The research team used the interview guides and narratives to identify areas, or themes, that were consistently investigated during data collection. The idea was to organize themes according to the dimensions in the RE-AIM framework to make it easier to compare themes across programs. For example, data from one case study indicated that the theme of *adapting intervention materials provided by the state or tribe to local community* is common to several local sites; the theme is categorized as an *implementation* dimension within the RE-AIM framework.

#### Identifying practices of interest

Identifying practices of interest is a multistep process: 1) select the practice within each theme that may be a best practice, 2) determine which sites use the practice of interest, 3) determine the purpose of the practice, 4) determine whether the purpose varies across sites, and 5) develop simple categories of the purpose (if applicable) that may help discern what makes it useful.

To illustrate this process, we use the example identified above: *adapting intervention materials provided by the state or tribe to local community*. In this example, we determined that some, but not all, local sites within the state or tribal project adapted intervention materials provided by the state or tribe to fit the needs of their community (steps 1 and 2). We found that the purpose of these adaptations varied, as did the actual adaptations (steps 3 and 4). By examining data in this way, we could assess which local sites adapted state or tribal materials and the purpose of the adaptation. We then could categorize the purposes (step 5). We learned that two sites adapted materials provided by the state by simplifying them and making them more accessible to and flexible for participants. The third site made no adaptations.

#### Applying a best-practices algorithm

**Figure 5:**
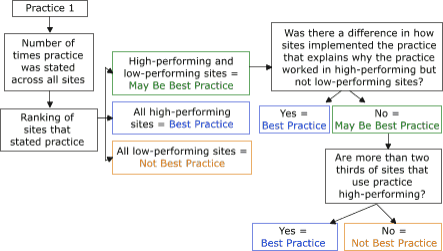
Algorithm for determining best practices in selected WISEWOMAN programs.

Before the final step in the analysis, we obtained the rankings for each local site. This was necessary if we were to select the best practices from those identified as a practice of interest in step 3. To make the selection, we developed an algorithm to tabulate the qualitative data by applying standardized decision rules to the information (Figure 5). Following the algorithm, we determined how many times the practice was observed and then assessed the ranking of the sites that used the practice (i.e., high-performing or low-performing). The ranking told us how well sites are reaching desired program outcomes, averaged across multiple indicators.

When only high-performing sites were using a practice of interest, it was considered *best*. To allow innovative practices at a single site to be eligible for best practices, and to allow the practices in a small number of sites to be eligible, a practice was considered *best* if all the sites using it were high-performing, regardless of whether one or 10 sites were using it. In contrast, when only low-performing sites were using a practice, it was not considered a best practice.

It was more difficult to identify a practice as best when it was being used by both high-performing and low-performing sites. In such cases, we attempted to learn how implementation of the practice differed at each site to assess whether the higher-performing sites were doing something different to contribute to their better outcomes. If we found a difference and could identify the factor that made the difference, we considered the practice to be a best practice. If both types of sites were using a practice, and there was no obvious difference in how it was used, then we deemed the practice *best* if more than two thirds of the sites were high performing. This ratio is consistent with the ratio of high-performing and low-performing sites selected for each project area (i.e., two high-performing, one low-performing). If only one high-performing and one low-performing site used a practice, the practice was not considered best because only half who used the practice was high performing.

This logic should be used with caution for the following reason: a composite score was judged to be the best overall indicator of a site's performance because it does not overemphasize the importance of any one RE-AIM dimension. In the algorithm step, however, we judged the effectiveness of certain site practices on the basis of these composite scores of site performance. For this reason, practices identified as best may not always lead to the desired outcomes. The success of a practice also may depend on the context in which it was implemented. To this end, the toolkit will provide information about the context in which those best practices were implemented.

## Results

### Lessons learned in developing and implementing the methodology

Piloting the methods in one project before collecting data from the other four projects was extremely helpful in refining our data collection instruments and methods. Data collected during the pilot using the initial protocols was overly process oriented. We realized that we needed to revise our methods to target program practices related to the lifestyle intervention. We also learned that conducting preliminary interviews with program staff provided us with an initial understanding of how the project or local site operated and helped us identify information we needed to collect onsite. The interview helped to build rapport with staff and made time available during site visits to ask more detailed questions about how and why practices were implemented.

The pilot also revealed the importance of observing a lifestyle intervention session at each local site because it gave us a much better understanding of how the intervention was actually implemented. Following the pilot, observing the lifestyle intervention became a key component of data collection in the remaining case studies. When arranging a visit to a local site, we selected and coordinated the day's activities around the observation, resulting in a greater probability of completing this piece of data collection. Nevertheless, it was a challenge to arrange the observations, particularly given privacy and confidentiality concerns. To address confidentiality concerns, we e-mailed the IRB approval of the observation protocol, the focus group guide, and the consent form to each site. We also allowed at least one month for planning a visit so we could work through any problems that arose. During this planning phase, we e-mailed a detailed list of the case study components to the sites and reviewed the components — including the observation — with them on the telephone.

Another methodological issue we encountered was difficulty in recruiting women for focus groups. Recruitment of women was especially challenging at sites that served a large geographic area: transportation over long distances was an issue for many women. Some sites recruited participants themselves because they were concerned that providing us with telephone numbers would breach confidentiality. We preferred this method because local site staff typically had a rapport with their participants. However, sites were not always able to help with recruitment because of time constraints. Advance planning was key to successful recruiting. Despite the challenges involved, the focus groups were worth the effort; they were vital for giving a voice to program participants.

### Dissemination

Disseminating the best practices in a toolkit is a critical final step in this evaluation. The process for developing this toolkit began by conducting a needs assessment with staff from currently funded WISEWOMAN projects during their 2004 annual meeting. Through this needs assessment, we learned not only about the kinds of information that projects would find most useful but also about the formats in which the material would be accessible to projects and local sites. The result will be a toolkit that provides a portfolio of ideas and options that current and newly funded WISEWOMAN projects can use. It will present concrete information on best practices for delivering the lifestyle intervention, including the specific mechanisms used by projects or sites to develop the interventions, the challenges they faced, and other issues relevant to the replication or adaptation of these practices. The toolkit also will describe the context or contexts in which each best practice was implemented; the idea is to acknowledge and let others know that some practices work in some settings but not in others. We expect the toolkit to be completed in spring 2006.

## Discussion

By using a mix of quantitative and multiple qualitative methods in our study of WISEWOMAN programs, we minimized the weaknesses inherent in each method and improved the completeness, and therefore the applicability and quality, of the data collected. By triangulating data collected from different sources, we were able to develop a more complete picture of how programs implement the lifestyle intervention and to identify best practices in implementation. The data on practices is detailed and complete enough to provide guidance for other WISEWOMAN programs interested in replicating the practices. A mixed-methods approach is clearly a promising strategy for identifying best practices in current programs. We recommend that others interested in using this method consider the strengths and weaknesses associated with each mode of data collection before settling on an approach. We also strongly encourage researchers to pilot their methodology.
