# Supplementary figures and images for: Identifying Best Practices for WISEWOMAN Programs Using a Mixed-Methods Evaluation
Source: Prev Chronic Dis. 2005 Dec 15;3(1):A07. (PMC1500960)

**Figure 1.** Site-selection method for WISEWOMAN study on best practices using the RE-AIM framework.

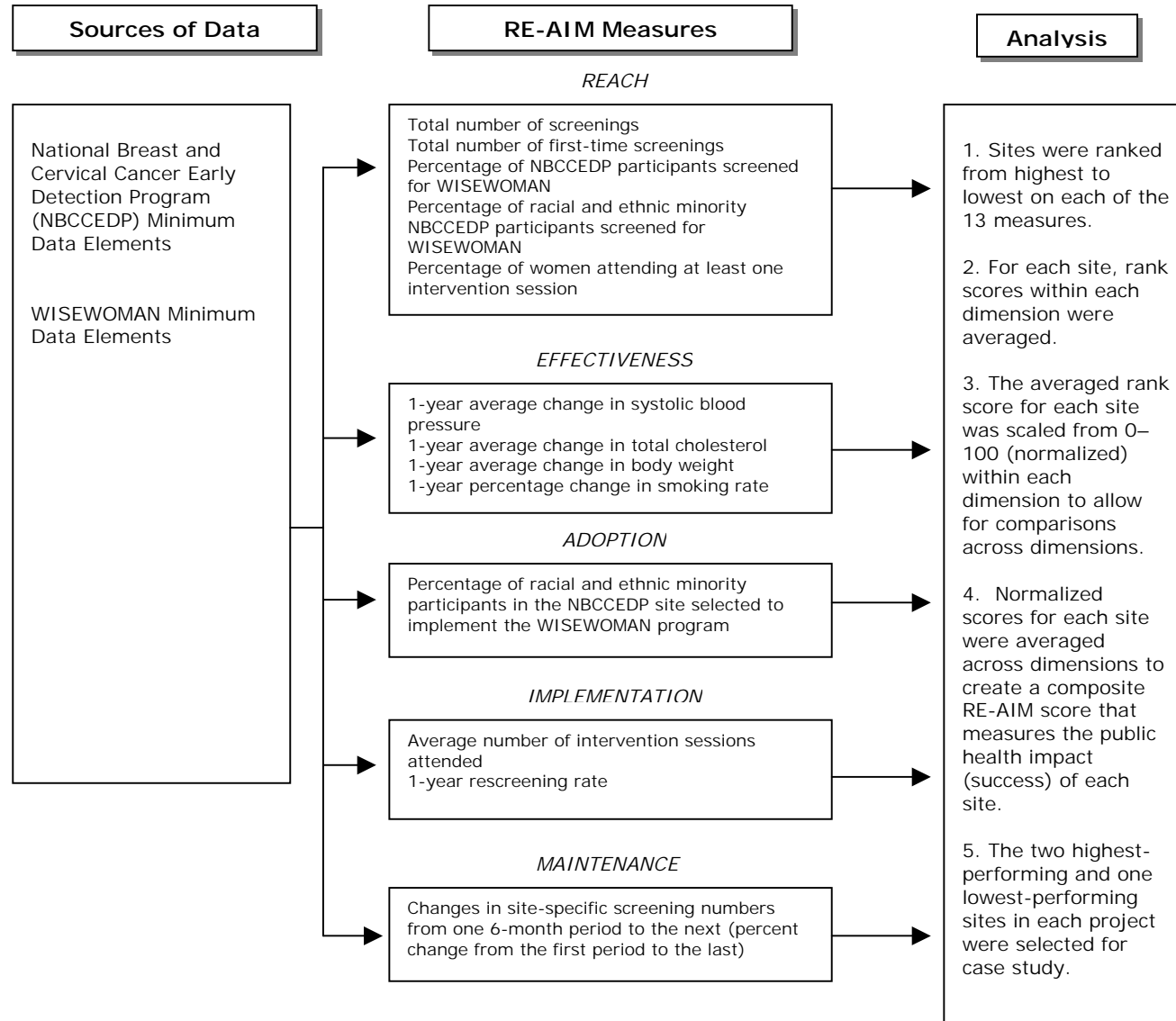

Supplement: Supplementary file 1 [file 05_0133_01.pdf]

**Figure 3.** Framework for guiding qualitative inquiry for WISEWOMAN study on best practices.

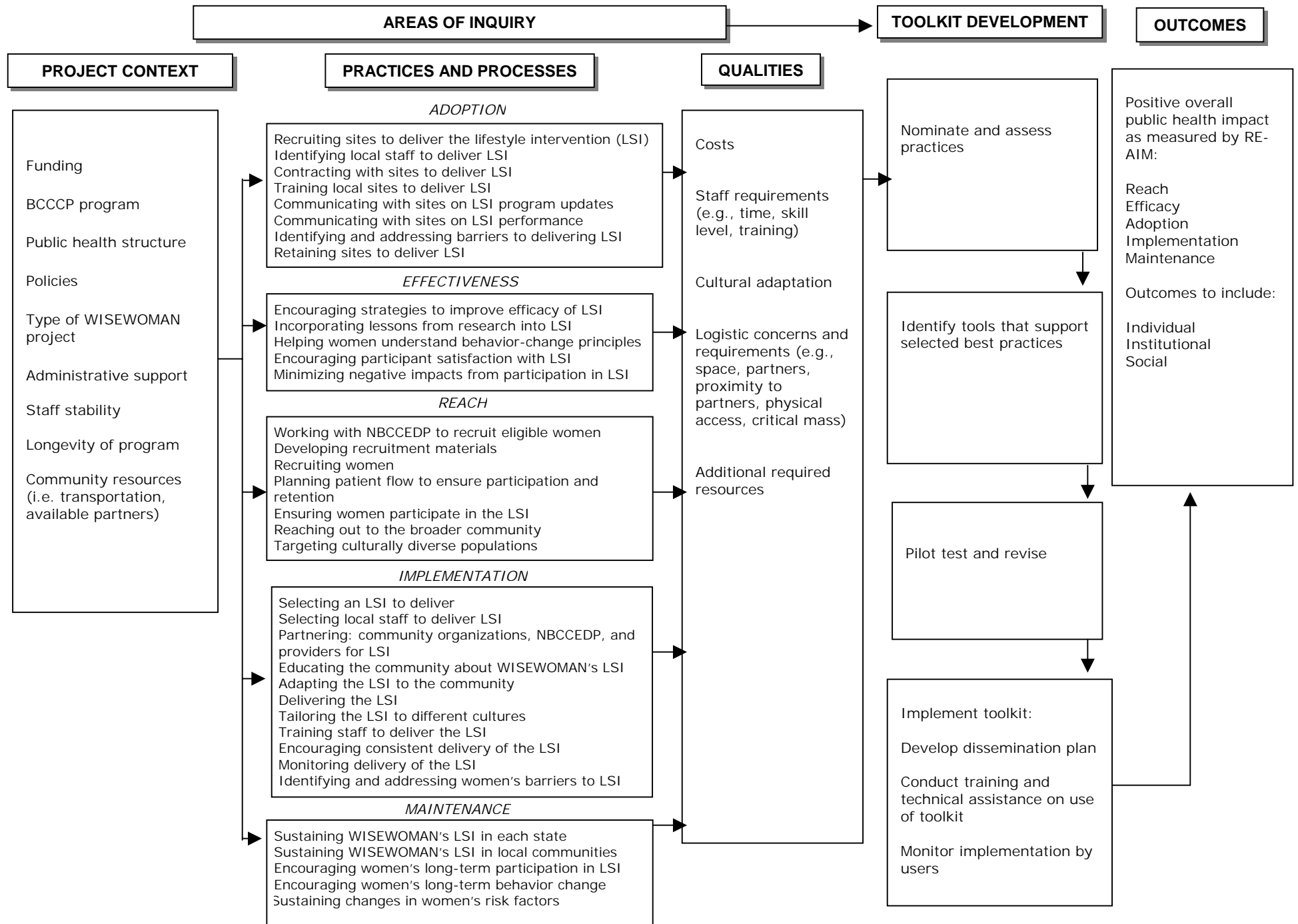

Supplement: Supplementary file 2 [file 05_0133_03.pdf]
